# Early detection of recurrence by ^18^FDG-PET in the follow-up of patients with colorectal cancer

**DOI:** 10.1038/sj.bjc.6604263

**Published:** 2008-02-26

**Authors:** I Sobhani, E Tiret, R Lebtahi, T Aparicio, E Itti, F Montravers, C Vaylet, P Rougier, T André, J M Gornet, D Cherqui, C Delbaldo, Y Panis, J N Talbot, M Meignan, D Le Guludec

**Affiliations:** 1 Université Paris 12 et Hôpital Henri Mondor, 51 Av du Mal de Lattre de Tassigny, Créteil 94100, France; 2 Hôpital Saint Antoine, Paris 75012, France; 3 Hôpital Bichat Claude Bernard, Paris 75018, France; 4 Hôpital Tenon, Paris 75020, France; 5 Hôpital Ambroise Paré, 92 Boulogne, France; 6 Hôpital Saint Louis, Paris 75010, France; 7 Hôpital Lariboisière, Paris 75010, AP-HP, France

**Keywords:** FDG PET, CT scan, colon cancer, follow up

## Abstract

We assessed the potential benefits of including systematic ^18^fluorodeoxyglucose positron emission tomography (FDG-PET) for detecting tumour recurrence in a prospective randomised trial. Patients (*N*=130) who had undergone curative therapy were randomised to undergo either conventional (Con) or FDG-PET procedures during follow-up. The two groups were matched at baseline. Recurrence was confirmed histologically. ‘Intention-to-treat’ analysis revealed a recurrence in 46 patients (25 in the FDG-PET group, and 21 in the Con group; *P*=0.50), whereas per protocol analysis revealed a recurrence in 44 out of 125 patients (23 and 21, respectively; *P*=0.60). In another three cases, PET revealed unexpected tumours (one gastric GIST, two primary pulmonary cancers). Three false-positive cases of FDG-PET led to no beneficial procedures (two laparoscopies and one liver MRI that were normal). We failed to identify peritoneal carcinomatosis in two of the patients undergoing FDG-PET. The overall time in detecting a recurrence from the baseline was not significantly different in the two groups. However, recurrences were detected after a shorter time (12.1 *vs* 15.4 months; *P*=0.01) in the PET group, in which recurrences were also more frequently (10 *vs* two patients) cured by surgery (R0). Regular FDG-PET monitoring in the follow up of colorectal cancer patients may permit the earlier detection of recurrence, and influence therapy strategies.

Colorectal cancer (CRC) is the second most common cause of cancer-related deaths in Western countries. Most newly-diagnosed cases already have a tumour invading across the bowel wall with lymph node invasion (stage III disease) and/or synchronous metastases (stage IV). Treatment is based on curative surgical resection (R0). However, approximately half of the patients who undergo curative R0 surgery go on to develop recurrent disease, and the median survival time after the operation is no more than 2 years (Griffin *et al*, 1987; Rodriguez-Moranta *et al*, 2006). Adjuvant chemotherapy improves prognosis in these patients, but more than one-third of them still experience recurrence within the 2 years following curative therapy (Moertel *et al*, 1995; Mitry *et al*, 2005; Van Cutsem and Costa, 2005). The preferred treatment for patients with recurrent disease is resection of the metastases in the liver or lung, and this can result in a 5-year survival rate of 35–40% (Scheele *et al*, 1990; Fong *et al*, 1997). This is why these patients should be followed-up using either clinical or biological exams, as well as imaging procedures (Desch *et al*, 2005; Rodriguez-Moranta *et al*, 2006; Sjovall *et al*, 2007).

Positron emission tomography (PET) using ^18^fluorodeoxyglucose (FDG) has emerged as a promising diagnostic imaging method in reassessing recurrent colorectal cancer, and can potentially improve the selection of patients for surgery, and hence may have a beneficial impact on the outcome of treatment. ^18^Fluorodeoxyglucose positron emission tomography (FDG-PET) detects changes in glucose uptake and metabolism, and also provides information about the location of a cancer within tissues. It is now considered to be a sensitive and accurate technique, and several studies have suggested that it should be carried out before resection of liver metastases from CRC (Stokkel *et al*, 2001; van der Hiel *et al*, 2001; Fernandez *et al*, 2004; Truant *et al*, 2005; Wiering *et al*, 2005; Khan *et al*, 2006).

However, these studies focused mainly on the diagnoses obtained with FDG-PET, and most of them included only a retrospective analysis taking clinical management decisions into account. As far as we know, only one open pilot study has carried out a prospective analysis of the impact of FDG-PET in patients already undergoing conventional management, and who were potentially eligible for resection of colorectal liver metastases (Ruers *et al*, 2002). Although CT scanning is the most common imaging procedure used to follow-up patients, FDG-PET is now sometimes used in patients with colon or rectal cancer. We do not know whether FDG-PET provides a more accurate assessment of the cancer stage than CT-scanning. This study was intended to assess the contribution of systematic FDG-PET to the detection and treatment of CRC recurrence following curative surgery in patients with a high risk of recurrence.

## PATIENTS

Between January 2001 and June 2004, 130 patients from seven teaching hospitals underwent curative R0 surgery for colon or rectal cancer. They were routinely assessed prospectively at regular 3-monthly intervals up to 24 months after curative surgery, or until death.

## METHODS

**Figure 1:**
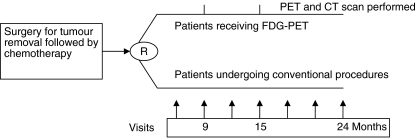
Study scheme.

At the second visit after curative surgery (6-month follow up visit), compliance with adjuvant chemotherapy, and the absence of disease progression and/or missed synchronous metastases were checked. Patients were randomly divided into two groups: one group received a conventional work-up (Con) and the other underwent PET. The baseline date was that of the initial surgery. The study follow-up started from the ninth month after baseline, and continued until the twenty-fourth month or the patient's death. All patients gave informed consent for the study, which comprised six visits (see schedule in Figure 1), a physical examination, biomarker assays (serum CEA or CA19-9, or both), an ultrasound scan (US) every 3 months (except after 9 and 15 months of follow-up), a chest X-ray every 6 months, and abdominal CT scans after 9 and 15 months of follow-up. Patients in the PET group also underwent ^18^FDG-PET after 9 and 15 months. Various study end points were recorded for the patients: the overall rate of recurrence in each group after 15-months follow up, the time until a recurrence was detected by at least one of the procedures described above, the time to second-line surgical intervention and/or drug treatment, including either chemotherapy or palliative therapy and the overall rate of curative surgery, if any, in each study group.

Biological marker assays as well as US and CT scans were performed in unselected laboratories by unselected biologists and radiologists in routine experiments. ^18^FDG-PET was performed by selected, trained nuclear physicians using the same machine (at the Tenon Hospital). Physicians were unaware of the findings of the CT-scan. Patients fasted for at least 6 h before the PET examination. Patient serum glucose levels were checked to ensure that they were in the normal range. An FDG solution, with an activity of 2 MBq per kg of body mass (corresponding to 150±20 MBq for an average adult), was then infused intravenously. PET imaging was performed using a C-PET tomograph (Philips, Eindhoven, The Netherlands) in three-dimensional acquisition mode, and with an axial field of view of 25 cm. The spatial resolution was 4.0 mm full-width at half-maximum. An emission image and transmission image were recorded at each bed position for 6 and 1 min, respectively, beginning 60 min after a tracer injection. The field of view extended from the base of the skull to the subinguinal region, requiring six to seven bed positions. The iterative reconstruction programme was run under X-Windows with a Motif user interface on a Sun workstation (SUN Microsystems, Palo Alto, CA, USA). Coronal, sagittal and transaxial images were produced from emission data with and without attenuation correction, based on an ordered subsets-expectation maximisation (OSEM) iterative reconstruction algorithm. Two experienced nuclear medicine specialists interpreted the slides, and reported whether they thought a recurrence was present (Yes/No), taking into account the patient's history, and the findings of the standard imaging procedures (not including CT). This yes/no response was classified as a correct or incorrect diagnosis according to the standard guidelines for recurrence, as follows. Recurrence was identified from histological samples (from biopsy or curative surgery) in all cases except in those with evidence of recurrence consisting of disseminated metastases or those for whom clinical examination, tumour markers and imaging procedures (routinely discussed during a multidisciplinary staff meeting) yielded consistently positive results. Patients requiring radiofrequency ablation of hepatic lesions underwent biopsy for histological analyses before treatment was started. Any discrepancy between the FDG-PET findings, and those obtained by other imaging procedures or a physical examination, were taken to be indicative of recurrence, and this was confirmed by a biopsy. There were several options in cases in which the FDG-PET results were consistent with recurrence: (i) continuing with surgery if the image showed one or a few localised lesions; any additional examinations required by the surgeon could be performed before surgery, (ii) biopsy of rectal, colonic, peritoneal, liver, pulmonary or nodular lesions, (iii) chemotherapy and/or palliative care if required for multiple recurrent tumours. All imaging findings were correlated with the subsequent final histological diagnosis, based on findings at surgery and/or from biopsies.

A new CT-coupled PET machine (PET-CT) became available from July 2004. This machine provided more effective PET/CT imaging than the first generation PET scanner; we therefore stopped recruiting patients to the study after including the one hundred and thirtieth patient. This was based on ethical concerns (the chances of patient survival with PET alone *vs* PET-CT) and methodological considerations (consistent imaging quality in all patients).

### Statistical analysis

The primary end point was to detect recurrence after 9 and 15 months of follow up in each group, on the basis of the intent-to-treat principle, that is, data from all randomised patients were analysed according to the strategy group to which they had been assigned when randomised. We assumed that the overall recurrence rate over the 2-year follow-up period would be similar in the two groups, but that recurrences might be detected earlier in the PET group. Assuming an overall estimated 30% recurrence rate, 30 patients with a recurrence need to be included in each group. By assuming a 30 percent difference in curative treatment for recurrence between the two arms (an estimated absolute gain of 10% in Con arm, and 40% in PET arm; *α*=5% unilateral test and *β*=20%), this requires 180 patients (90 in each arm) over a 2-year follow-up period. We compared the number of patients diagnosed as having a recurrence, the time to onset of recurrence after curative surgery, and the time until second-line therapy was started after the confirmed diagnosis of recurrence. Continuous variables were expressed as mean±s.d., and were compared using Student's *t*-test. Differences in qualitative variables were evaluated using *χ*^2^ test, or the Fisher's exact test when necessary. We used the Kaplan–Meier survival analysis and the log-rank test to describe and compare the time until recurrence or second-line therapy in the two groups of patients (PET and Con). All patients with malignant tumours detected during the follow-up were pooled, and analysed as a single recurrence group. Patients who died and in whom no recurrence had been detected were considered to have been in remission with progression-free survival until the time of their death. In other cases, the progression-free survival period lasted until the date of recurrence or the date of the patient's death. The overall rates for sensitivity, specificity, positive predictive value and negative predictive values for the CT and PET strategies were calculated on the basis of the recurrences documented during the 2-year follow-up period.

## RESULTS

**Figure 2:**
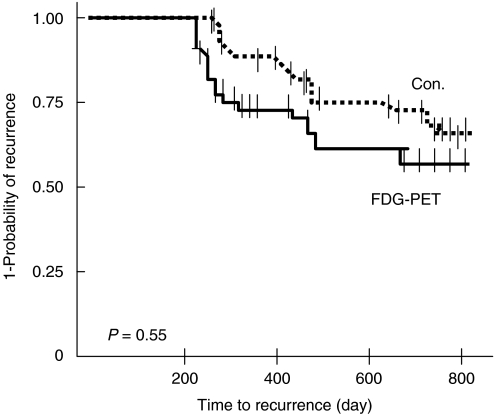
Kaplan–Meier curve for time to recurrence detected in patients with curative therapy for colon or rectal cancer.

**Figure 3:**
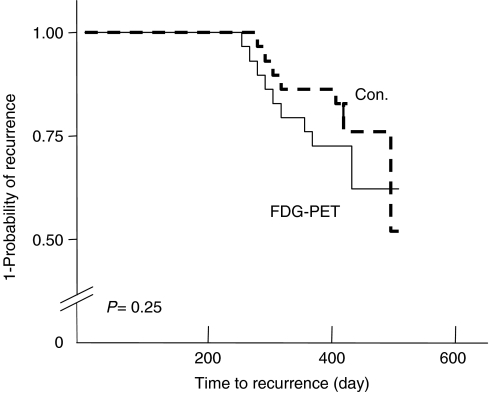
Kaplan–Meier curve for time to recurrence in asymptomatic patients without elevated tumour marker levels.

One hundred thirty patients (65 in each group) were evaluated in an ITT analysis; five were excluded from the PP analysis because of missing data. The two arms were matched with regard to the characteristics of both the patients and tumours (Table 1). Overall, the number of patients with a detected recurrence was 44 (23 in the PET group and 21 the Con group; *P*=0.60). Recurrence was confirmed either by biopsies or surgery in 27 (21.6% in the PP analysis) of these 44 patients: 15 out of the 60 (25%) patients in the PET-group, and 12 out of the 65 (18.5%) patients in the Con group (*P*=0.19). Kaplan–Meier curves for the time from baseline until the detection of a recurrence of the disease during follow-up were obtained, and ITT analysis performed (Figure 2). There was no significant difference between the PET and Con groups with regard to actuarial curves of recurrence (log-rank, *P*=0.55); however, for all the patients with a recurrence, the time from baseline until detection of the recurrence was significantly shorter (*P*=0.01) in the PET group (12.1±3.6 months) than in the Con group (15.4±4.9 months). However, if we consider only asymptomatic patients without elevated serum tumour markers, then a recurrence was detected in 34 patients (20 PET group patients and 14 in the Con group) by imaging procedures (CT, PET, Chest X-Ray, US). In this case, the time from baseline until the detection of a recurrence was shorter (although not significantly so) in the PET group than in the Con group (log-rank test, *P*=0.25) (Figure 3).

### Impact on therapy management

As specified in the study design, a curative surgical tumour resection procedure was performed in 17 out of 44 patients (PP analysis): two in the Con arm and 15 in the PET arm (Table 2). In 11 patients, additional imaging procedures were needed before a final decision to treat could be reached: four in the PET group and seven in the Con group. An ^18^FDG-PET scan was performed in two patients in the Con arm, because despite elevated tumour marker levels, no evidence of recurrence was found using Chest X-ray, CT Scan and US. Curative R0 surgery (Table 3) could be performed in 12 cases, more frequently (*P*<0.01) in patients in the PET group (10 out of 23; 43.5%) than in those in the Con group (two out of 21; 9.5%). It is interesting that the FDG-PET examination was the only imaging procedure to provide a positive finding in six (out of 10) patients in the PET arm, whereas these recurrences were also detected by conventional imaging procedures in the other four patients. In five out of 17 patients who underwent surgery for curative resection, subsequent pathology reports identified a second neoplasm in three cases (one case of gastric GIST, and two cases of primary pulmonary tumours), and no tumour could be found in two cases (in one case, a left liver lobectomy revealed a sarcoidosis, and in the other the pathology findings identified an inflammatory infiltrate in the pelvic floor). Even if the detection of a second malignant tumour was not considered to be a ‘recurrence’ in the PP analysis for times from baseline until detection, there was no significant difference between PET and Con patient groups (log-rank, *P*=0.61). Chemotherapy was administered in 39 cases (19 and 20), and palliative therapy in five cases (two and three) in the PET and Con groups, respectively. Overall, curative surgery (R0) was performed or a new course of chemotherapy was started sooner after baseline in the PET group (14.8±4.1 months) than in the Con group (17.5±6 months; *P*=0.09). At 24 months, nine out of 44 patients had died: three in the PET-group, and six in the Con group (Table 2 and 3).

### PET reports compared to all other procedures

Taking all the patients who had undergone an ^18^FDG-PET, positive results were recorded in 28 out of 67 patients: (i) a recurrence of colon or rectum cancer was detected in 22 patients (20 in the PET group and two in the Con group), (ii) additional primary cancers were found in three patients, all of them in the PET group; (iii) false-positive results were recorded for three patients, all in the PET group, leading to non beneficial procedures (surgery and/or additional imaging). Conversely, FDG-PET failed to detect peritoneal carcinomatosis that was subsequently confirmed by laparoscopy in two patients in the PET group; in one case the patient had rising serum tumour marker levels, and in the other, high protein levels and abnormal peritoneal fluid cytology. The rate of false-negative results was 8% (two of 25) in the PET group. However, in the Con group, recurrences were correctly identified in two patients using ^18^FDG-PET, and in the PET group recurrences were detected in four patients using ^18^FDG-PET as the only imaging procedure. Thus, the overall rates of sensitivity, specificity, positive predictive value, negative predictive values for detecting recurrence were 91, 93, 88.6 and 95% respectively in the conventional arm, and 96, 92.1, 89.2 and 97.2%, respectively in the PET arm.

## DISCUSSION

This is the first prospective controlled randomised study using FDG-PET to monitor patients with stage III or IV colon or rectal cancer. We show that FDG-PET is a valuable adjunct to conventional follow-up in patients with a higher risk of recurrence, including those who may be candidates for resection of colorectal, liver or lung metastases. One-third of our patients experienced a recurrence during a 2-year follow-up period, and the rate of curative surgery for liver and/or pulmonary metastases resection was higher in the PET group patients than in the Con group patients, probably due to earlier detection.

Recurrence of colon and rectal cancer may occur in asymptomatic patients, and this means that physical examinations, biological marker assays and imaging procedures must also be performed (Figueredo *et al*, 2003; Meyerhardt and Mayer, 2003). However, improvements in the survival of patients who have had curative therapy for colon or rectum cancer have been attributable to intense follow-up programmes including imaging procedures (Northover *et al*, 1994; Figueredo *et al*, 2003). Among these techniques, CT scans seem to be more sensitive than US for detecting liver metastases (Pietra *et al*, 1998; Schoemaker *et al*, 1998; Secco *et al*, 2002), and this procedure is now recommended (Benoist *et al*, 2006), although 3–15% of recurrences may still be missed (Holt *et al*, 2006). Thus, the possibility of performing R0 curative surgery if the only liver imaging performed is CT scanning appears to be limited. An accurate imaging procedure for the detection of lung metastases is also necessary. although, CT of the chest did not appear to be the most cost-effective procedure (Goldberg *et al*, 1998).

Nevertheless, detection of a recurrence should lead to curative therapy with an aggressive surgical approach. This requires the early detection of recurrences in asymptomatic patients whether tumour marker levels are elevated or not. In this study, adding systematic FDG-PET to the routine follow-up procedures resulted in a higher number of curative surgical interventions being performed in the PET group than in the Con group. This significant difference is probably due to the delayed detection of recurrence and the additional examinations required to support decisions about therapeutic strategy in the Con group, and to the detection of three additional primary cancers in the PET group. These were also consistent with the findings of another prospective uncontrolled study in which ^18^FDG-PET was used for patient monitoring (Ruers *et al*, 2002), and in which there were two times as many deaths in the Con group as in the PET group. Although, our findings suggest that combining the use of whole-body FDG-PET with conventional anatomic imaging may significantly improve patient survival, the number of patients investigated does not allow us to reach a firm conclusion about this.

Recent PET machines have a higher detection rate for small tumours than the machine used in this study, and coupled PET-CT appears to provide more accurate diagnoses than performing the two examinations separately (Cohade *et al*, 2003; Even-Sapir *et al*, 2004; Kamel *et al*, 2004; Nakamoto *et al*, 2007). It permits a more accurate interpretation, and improves the detection of lesions. Thus, we would expect combined PET-CT to make it easier to determine the stage of the disease correctly. Further investigation with a greater number of patients and PET-CT procedure is required to find out whether the early detection of recurrences during follow-up screening is cost-effective.

In summary, using this new follow-up strategy increased the rate of curative resection (R0) in patients by allowing us to detect CRC recurrences at an earlier stage. We would therefore expect improved patient survival if such a follow-up programme was undertaken. We now need to assess the cost-effectiveness of strategies including the systematic use of FDG PET/CT in patients who have developed stage III and VI colon and rectum cancer following curative surgery.

## Figures and Tables

**Table 1:** Patient characteristics

**Variables/patient group**	**FDG PET**	**Con**	***P***
Patients, *n*	65	65	—
Age, mean (year) (s.d.)	58.1 (11.2)	62.0 (12.1)	0.63
*Location*^a^ *of tumours*			
% Colon	56.2	59.4	0.86
% Rectum	43.8	40.6	
			
Differentiation** of the tumour (%)			
Good^a^	67.2	57.8	
Intermediate	1.6	4.7	
Poor	31.2	37.5	0.41
Stage IV (%)	12.1	13.8	0.16
			
*Neo-adjuvant treatment (%)* a			
Yes	11.1	13.8	
No	88.9	86.2	0.79
			
*Adjuvant treatment (%)* a			
Yes	90.5	89.2	
No	9.5	10.8	0.99
Time (day) since surgery, mean (s.d.)	231 (60.36)	223.7 (60.36)	0.69

Con=conventional, FDG-PET=^18^fluorodeoxyglucose positron emission tomography.

a 1 missing value.

^**^4 missing values.

**Table 2:** Recurrence (course and management) in patients with stage III and VI colon and rectal cancer during a 2-year follow-up strategy of conventional surveillance or additional PET-18FDG

	**Intention to**	**Per protocol**	***P***
Total, *N*	130	125	
FDG-PET	65	60	—
Con.	65	65	
			
*Recurrence, N*	46	44	
FDG-PET	25	23	—
Con	21	21	
			
*Time to recurrence (month) ±s.d.*			
FDG-PET	12.0±4.9	12.1±3.7	
Con	15.3±4.9	15.4±5	0.01
			
*Time to therapy (month)±s.d.*			
FDG-PET	15.5±5	14.8±4.1	0.09
Con	17.5±6	17.5±6	
			
*Biopsies and surgery*, *N* *(% over total)*	27 (20.8)	27 (21.6)	
PET-18FDG	15 (23)	15 (25)	
Con	12 (18.5)	12 (18.5)	0.67
			
*Surgery operation, N* *(% over recurrences)*	17 (37)	17 (38.6)	
PET-18FDG	15 (60)	15 (65)	
Con	2 (9.5)	2 (9.5)	<0.0001
			
*R0 curative, N* *(% over recurrences)*	12 (26)	12 (27.3)	
PET-18FDG	10 (40)	10 (43.8)	
Con	2 (9.5)	2 (9.5)	<0.01
			
*Death, N (% over recurrences)*		9 (20.5)	
FDG-PET	—	3 (13)	
Con	—	6 (28.5)	0.33
			
*Time to (months)*			
FDG-PET	—	8.4, 21.3, 23.2	
Con	—	9.1, 9.4, 10, 12, 13, 21.3	

Con=conventional, FDG-PET=^18^fluorodeoxyglucose positron emission tomography.

**Table 3:** Characteristics of patients undergoing R0 surgery for recurrence during a 2-year follow-up period in patients with rectal or colon cancer

	**Con arm**	**PET arm**	**Total**
Total, *N*	2^a^	10^a^	12
*pTNM staging at baseline*			
stage III	1	8	9
stage IV	1	2	3
			
*Site of recurrence*			
Abdominal, liver	2	6	8
Other	0	2	2
Thoracic, pulmonary	0	2	2
Time to therapy (month) ±s.d.	9.4 & 16.3	12.2±3.6	13.8±4.3
Death, up to the end of study	0	0	0

Con=conventional, PET=positron emission tomography.

a Additional imaging procedures including MRI or PET arm were needed to best characterise sites and numbers of metastasis.
